# Of Asian Forests and European Fields: Eastern U.S. Plant Invasions in a Global Floristic Context

**DOI:** 10.1371/journal.pone.0003630

**Published:** 2008-11-03

**Authors:** Jason D. Fridley

**Affiliations:** Department of Biology, Syracuse University, Syracuse, New York, United States of America; University of Zurich, Switzerland

## Abstract

**Background:**

Biogeographic patterns of species invasions hold important clues to solving the recalcitrant ‘who’, ‘where’, and ‘why’ questions of invasion biology, but the few existing studies make no attempt to distinguish alien floras (all non-native occurrences) from invasive floras (rapidly spreading species of significant management concern), nor have invasion biologists asked whether particular habitats are consistently invaded by species from particular regions.

**Methodology/Principal Findings:**

Here I describe the native floristic provenances of the 2629 alien plant taxa of the Eastern Deciduous Forest of the Eastern U.S. (EUS), and contrast these to the subset of 449 taxa that EUS management agencies have labeled ‘invasive’. Although EUS alien plants come from all global floristic regions, nearly half (45%) have native ranges that include central and northern Europe or the Mediterranean (39%). In contrast, EUS invasive species are most likely to come from East Asia (29%), a pattern that is magnified when the invasive pool is restricted to species that are native to a single floristic region (25% from East Asia, compared to only 11% from northern/central Europe and 2% from the Mediterranean). Moreover, East Asian invaders are mostly woody (56%, compared to just 23% of the total alien flora) and are significantly more likely to invade intact forests and riparian areas than European species, which dominate managed or disturbed ecosystems.

**Conclusions/Significance:**

These patterns suggest that the often-invoked ‘imperialist dogma’ view of global invasions equating invasion events with the spread of European colonialism is at best a restricted framework for invasion in disturbed ecosystems. This view must be superseded by a biogeographic invasion theory that is explicitly habitat-specific and can explain why particular world biotas tend to dominate particular environments.

## Introduction

Throughout history, plant and animal assemblages have evolved in isolated biotas that have occasionally come into contact with one another, and the resulting interchange has usually been the near-wholesale replacement of one region's species with another's [1]–[4]. Modern, human-assisted plant invasions are a clear analogue of historical biotic interchanges [3], [5], and yet relatively few invasion biologists have asked whether there are regular patterns of global dominance of plants from particular floristic regions [6], [7]. If such patterns exist, they would be of prime importance to the management community concerned with invasions, as resources for prevention and control could be focused on those regions most likely to be sources of future invaders [8]. Such patterns would also be a significant advance for ecologists and evolutionary biologists still struggling to identify generalizations concerning which plants invade [9], [10], [11] and which communities are most susceptible to invasion [12]–[15].

The Eastern U.S. (EUS) has seen an unprecedented spread of invasive species in nearly all major habitats over the past century [16], [17], and these non-native species represent nearly all of the world's floristic regions. The diversity of invader habitats and their provenances includes the turfgrass pest *Poa annua* (annual bluegrass, from Europe); the mesic forest understory grass *Microstegium vimineum* (Japanese stiltgrass, from southeast Asia); the floating freshwater aquatic *Eichhornia crassipes* (water hyacinth, from the Amazon basin); many fast-spreading shrubs of open, disturbed woodlands such as *Lonicera tartarica* (Tartarian honeysuckle, from the steppes of central Asia); major crop pests like *Cyperus rotundus* (purple nut sedge, probably from India); and canopy dominants that threaten to replace entire forest stands such as *Triadaca sebifera* (Chinese tallow tree). Is there any underlying pattern to which global floras contribute invaders to particular habitats? Are the provenances of those species that become invasive an unbiased subset of total alien flora, or are invaders more likely to come from particular evolutionary centers of origin?

To invade, a species must be introduced, establish, and spread [18], and processes specific to each of these stages could bias non-native floras toward particular provenances. For example, introduction attempts of non-native species to a focal region may vary according to geographic origin due to historical differences in rates of trade and travel between regions [19]. Furthermore, introduced species that become naturalized should preferentially come from areas that match certain climate, soil, or disturbance conditions that allow a species to reproduce without human assistance [20]. Finally, on top of floristic biases in both introduction attempts and naturalizations, species that become invasive—those that spread naturally and compete successfully with native vegetation—may preferentially come from certain regions where species have achieved superior levels of fitness under competition in a given environment, what Darwin [1] referred to as a “higher stage of perfection or dominating power”, and others have referred to as ‘preadaptation’ [21]. This hierarchy based on different mechanisms of introduction, establishment, and spread suggests that comparing floristic patterns of different components of non-native floras (e.g., the provenances of alien species versus the subset of those that become invasive) could help refine studies of biological attributes that allow a typically small subset of introduced species to become invasive. The hierarchy also suggests that non-native floristic associations should vary strongly by habitat type [3], [9], [22], [23], given 1) modes of introduction vary by habitat type, as accidental introductions are often agronomic and follow the spread of agricultural operations, whereas ornamental introductions span a larger range of potential environments (sun versus shade, xeric versus mesic); 2) global floristic regions vary greatly in habitat representation, and some floras lack major habitat types entirely (there is no mesic deciduous forest in the Sahara); and 3) superior competitive abilities are more important to invader success in some habitats, particularly those of low disturbance intensities [24], [25].

**Figure 1:**
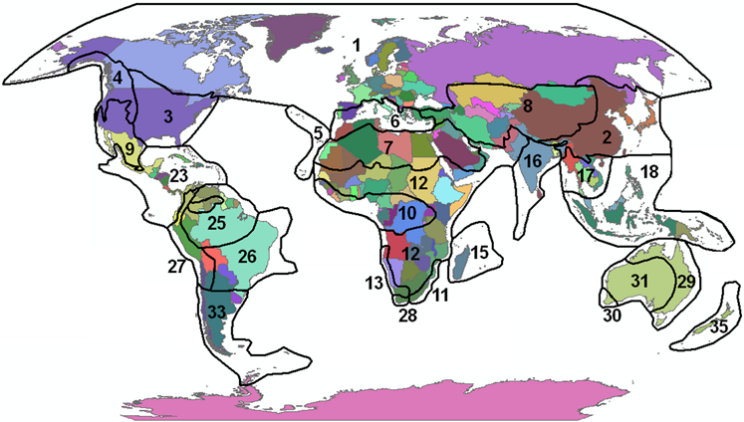
Floristic regions of the world, from Takhtajan [27]. Region names and associated statistics are listed in Tables 2 and 3. Those shown here do not include several largely oceanic or archipelagic regions ignored in the present analysis.

In this paper I analyze the alien and invasive vascular floras of the EUS coincident with the Eastern Deciduous Forest biome of North America [26] to determine whether alien and invasive plant species of this region are more likely to come from particular source floras, using the Takhtajan [27] global floristic regions as source areas that correspond to global centers of plant diversification (Fig. 1). Due to the prevailing view that strategies for plant success depend strongly on habitat qualities, which in turn suggests that global floras should preferentially contribute species to certain habitats, I conducted the analysis for invasive species using a habitat classification (Table 1) based on environmental differences that select for well known differences in plant strategies (disturbance regime and resource availability [25]). Two plant strategies associated with habitat type that are widely available for floristic-based analyses include species growth form (trees, forbs, etc.) and duration (annual, biennial, perennial); for these attributes I also asked whether native, alien, and invasive components of the EUS flora exhibit regular differences in attribute composition associated with floristic and habitat patterns. The primary objective of this study was to address whether modern plant invasions are qualitatively any different from biotic interchanges throughout the history of biotic migrations [4], [5], [28]—that is, whether biogeographic patterns of modern invasions reveal new evolutionary-based insights that provide a general framework for predicting where invaders come from and which areas are preferentially invaded.

**Table 1:** Habitat classes describing the environmental associations of plant invaders of the Eastern U.S.

Habitat	Description
Aquatic	Floating or submerged vegetation, in ponds, impoundments, lakes, or streams.
Forest	Habitats characterized by significant tree canopy cover, including woodlots, forests, suburban woodlands, open woodlands, disturbed forest, riverine woods, old homesites, wet forests, swamps, forested bottomlands, dry woodlands, ridgetop woods.
Managed	Unshaded habitat that is the product of continuing disturbance (annual or frequent basis), including agricultural systems (of turf, alfalfa, or other annual crops), pasture, rangeland, plantations, lawn, barnyards, gardens, cropland.
Open	Unshaded, early successional habitats that are the product of past or irregular natural or anthropogenic disturbance, including thickets, waste places, disturbed areas, old fields, sandy shores, hedgerows, fencelines, woodland edges, wood borders, fields, trails, urban lots, dunes, coastal sands, meadows.
Riparian	Habitats associated with flowing water, including riparian, streamside, stream banks, river banks, gravel bars, riverine forest, bottomland, floodplains, riverine woods, rivers, floodplain forest.
Roadside	Frequently disturbed habitat associated with transport, including roadsides, road banks, road ditches, right of ways, railroad embankments.
Wetland	Seasonally or continually wet terrestrial habitats, including wetland, seeps, ditches, bogs, marshes, lowlands, waterways.

Each invasive species was assigned to one or more classes based on habitat descriptions listed in major Eastern U.S. floras [52]–[54].

## Results

The alien flora of the EUS includes 2629 vascular plant taxa, 449 of which (17%) are documented as invasive (Table 2). Infraspecific taxa (subspecies and varieties) account for 304 of the alien taxa and 14 of the invasive taxa. Alien taxa of the EUS come from all major global floristic zones (Table 2). Nearly half (45%) of the alien taxa have native ranges that overlap the Circumboreal floristic region (including central and northern Europe; Fig. 1), followed in representation by the Mediterranean (39%), Irano-Turanian (31%), and Eastern Asiatic (24%) regions. Of the world floristic regions where nativity could be reliably assigned, the Neozeylandic region is the smallest donor to the EUS alien flora (7 taxa), and 5% of the alien taxa are derived from cultivation (many crops and ornamental plants). Of the 2629 taxa analyzed here, about 50 could not be reliably categorized into native floristic regions, due to lacking nativity information, highly questionable non-native status, or native-nonnative hybrid origin; eight of these were reported invasive (see Supplemental Dataset S1).

**Table 2:** Eastern U.S. alien and invasive floras categorized by global donor floristic regions [27].

Region	Region Name	Alien taxa	Invasive taxa	% Invasive	Alien taxa (region endemics)	Invasive taxa (region endemics)	% Invasive (region endemics)
1	Circumboreal	1196	266	22%	282	31	11%
2	Eastern Asiatic	622	180	29%	291	74	25%
5	Macronesian	303	53	17%	1	0	0%
6	Mediterranean	1027	205	20%	143	3	2%
7	Saharo-Arabian	105	44	42%	1	0	0%
8	Irano-Turanian	815	220	27%	68	2	3%
9	Madrean	42	8	19%	14	3	21%
10	Guineo-Congolian	93	11	12%	1	0	0%
11,13,28	[South African]	92	8	9%	6	0	0%
12	Sudano-Zambezian	189	20	11%	13	0	0%
15	Madagascan	52	6	12%	1	0	0%
16	Indian	147	26	18%	12	2	17%
17	Indo-Chinese	153	28	18%	4	2	50%
18	Malesian	149	22	15%	1	0	0%
23	Caribbean	96	9	9%	18	1	6%
25	Amazonian	66	10	15%	3	1	33%
26	Brazilian	157	14	9%	22	2	9%
27	Andean	99	8	8%	13	0	0%
29,30,31	[Australian]	103	9	9%	10	0	0%
33	Chile-Patagonian	132	11	8%	20	2	10%
35	Neozeylandic	7	0	0%	1	0	0%
	[Cultivation origin]	141	9	6%	NA	NA	NA
	*All Regions*	2629	449	17%	925	123	13%

Floristic regions refer to Fig. 1. Each region is listed with its contribution to the total alien and invasive flora of the Eastern U.S. Region endemics are those taxa native to a single floristic region.

**Figure 2:**
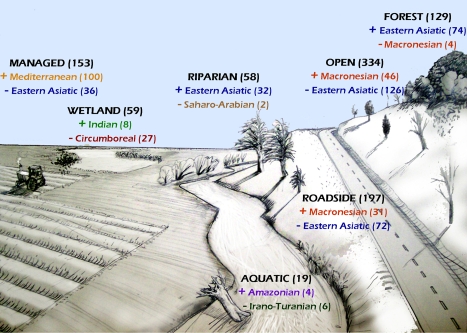
Floristic signature of Eastern U.S. plant invasions by habitat type. Seven habitat types (Table 1) are illustrated with the total number of species described as “invasive” (out of 449 total in the Eastern U.S.) listed in bold parentheses. Floristic regions most positively and negatively associated with each habitat were determined by the most extreme positive and negative standardized residual values from a Pearson chi-square test of a contingency table of all floristic regions and habitat types (Table 3). Number of invaders contributed to each habitat by each listed region are noted in parentheses. Drawing by Eric Fridley.

**Figure 3:**
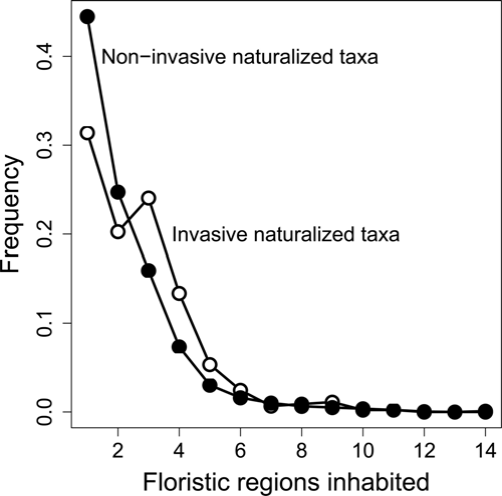
Relative frequency of the number of floristic regions inhabited by invasive and non-invasive alien plants of the Eastern U.S. Non-invasive taxa (N = 2180) are indicated by closed dots and invasive taxa (N = 449) by open dots. Candidate floristic regions (N = 21) are those listed in Table 2. A two-sample Wilcoxon rank sum test indicates invasive taxa span a larger range of native floristic regions than non-invasive taxa (W = 585242, P<0.001).

The subset of 449 invasive EUS taxa is not a random sample of native floristic regions of the alien taxa (Table 2, Fig. 2). Twenty-nine percent of the alien taxa with native ranges that include the Eastern Asiatic region are reported invasive, compared to 22% and 20% of the alien taxa from Circumboreal and Mediterranean regions. Alien taxa present in the Saharo-Arabian and Irano-Turanian regions also include high proportions of invasive taxa (42% and 27%, respectively). However, when alien taxa were instead restricted to those that only occur in a single native region (region endemics), the amount of invasive taxa from Saharo-Arabian and Irano-Turanian regions essentially disappeared (0% and 3%, resp.), as did those from the Mediterranean (2%). This in part reflects the clear relationship between native range size, measured as the number of floristic regions inhabited, and invasion potential (Fig. 3). Despite the smaller overall invasive proportion of region-endemic alien taxa (13%; Table 2), endemics from East Asia have nearly as high an invasive percentage as non-endemics (25%), whereas the percentage of endemic invaders from the Circumboreal region is cut in half (11%, compared to 22% non-endemic invaders). The Neozeylandic region is the only region to lack any invasive contribution to the EUS flora.

A majority of the invasive flora (74%) is found in open habitats of irregular disturbance, followed by roadsides (44%), managed (annually disturbed) ecosystems (34%), forests (29%), and wetland, riparian, and aquatic systems (13%, 13%, and 4%, respectively; Table 3). More than half of the woody invasive taxa (58%) are from the Eastern Asiatic region, and significantly more of the East Asian taxa (56%) are woody than expected based on the overall representation of woody invaders. At the other extreme, the invaders of four regions were significantly more likely to be herbaceous than the overall invasive pool, including those from the Sudano-Zambezian (100% herbaceous), Macronesian (96%), Saharo-Arabian (86%), and Mediterranean regions (80%; all P<0.05; Table 3).

**Table 3:** Habitats and growth forms that characterize the invasive plant species from each global donor floristic region.

Region	Region Name	Open	Managed	Forest	Wetland	Roadside	Riparian	Aquatic	Woody	Herbs	% Woody invaders
1	Circumboreal	208	113	61	27	132	28	11	70	203	26%
2	Eastern Asiatic	126	36***	74***	27	72	32*	6	101***	90	56%
5	Macronesian	46	23	4**	6	31	3	3	2	51***	4%
6	Mediterranean	166	100**	42	24	105	23	9	41	169***	20%
7	Saharo-Arabian	36	22	7	4	25	2	4	6	39**	14%
8	Irano-Turanian	180	89	58	26	114	29	6*	64	164	29%
9	Madrean	8	6	0	0	6	0	0	1	8	13%
10	Guineo-Congolian	8	6	1	2	5	1	1	0	11	0%
11,13,28	[South African]	3	2	0	1	3	1	1	0	8	0%
12	Sudano-Zambezian	14	8	1	4	10	4	2	0	20***	0%
15	Madagascan	4	3	0	0	4	0	1	0	6	0%
16	Indian	22	9	8	8	11	7	1	6	21	23%
17	Indo-Chinese	19	6	9	6	14	4	1	10	19	36%
18	Malesian	19	7	7	5	13	5	0	6	17	27%
23	Caribbean	7	3	1	0	4	0	1	3	8	33%
25	Amazonian	6	2	0	1	1	0	4	3	9	30%
26	Brazilian	9	3	0	2	4	0	4	6	11	43%
27	Andean	6	2	0	0	3	0	2	3	7	38%
29,30,31	[Australian]	7	1	3	3	4	3	1	4	7	44%
33	Chile-Patagonian	6	4	0	1	4	0	3	4	9	36%
35	Neozeylandic	0	0	0	0	0	0	0	0	0	0%
	[Cultivation origin]	5	4	0	1	2	1	0	5	4	56%
	*All Regions*	334	153	129	59	197	58	19	175	293	39%

Floristic regions refer to Fig. 1. Bold counts are statistically significant outliers in Pearson chi-square analysis with significance level indicated by asterisks (overall habitat x region χ^2^ = 243.6 on 120 df; P<0.001; growth-form x region χ^2^ = 104.1 on 20 df; P<0.001). *P<0.05; **P<0.01; ***P<0.001.

Several regions exhibited significant habitat bias in their invasive representation (Table 3, Fig. 2). Annually disturbed, managed ecosystems are far more prone to invasion from Mediterranean plants than plants from the Eastern Asiatic region (P<0.001; Fig. 2). Conversely, 41% (74/180) of the invasive taxa from East Asia invade forests, compared to only 7% (4/53) invaders from Macronesia and 29% of the invasive taxa overall (Table 3). Significant deviations in habitat representation among invaders from different source floras also include a greater representation of East Asian taxa in riparian habitats and very few Irano-Turanian species in aquatic habitats (Table 3). Major floristic patterns of the invasive pool of all habitat types are illustrated in Fig. 2.

The composition of species growth form and duration is significantly different among native, alien, and invasive EUS floras, with departures being greatest between the invasive and alien pools (Table 4). Compared to the native flora, the alien flora is overrepresented by annuals, biennials, and vines, and underrepresented by shrubs and grasses. Other growth form categories have remarkably similar representation in the native and alien floras, including the overall split between woody and herbaceous taxa (about 1 woody species in 5). In contrast, the growth form and duration composition of the invasive flora shows a strong departure from the alien flora. Invaders were significantly more likely to be perennial trees, shrubs, and vines, and thus much less likely to be herbaceous (65%) than both the native or alien floras (Table 4).

**Table 4:** The composition of native, alien, and invasive vascular plant floras of the Eastern U.S. with respect to growth habit and duration [46].

	Native flora		Alien flora		Invasive flora	
	# taxa	%	# taxa	%	# taxa	%
Trees	463	8%	243	9%	82***	18%
Shrubs	1149	21%	455***	17%	129***	29%
Vines	195	3%	177***	7%	**42** *	9%
Graminoids	1110	20%	411***	16%	54**	12%
Forbs	3436	62%	1720	65%	239***	53%
Woody	1330	24%	607	23%	175***	39%
Herbaceous	4546	82%	2131	81%	293***	65%
Annuals	1057	19%	985***	37%	122***	27%
Biennials	191	3%	228***	9%	45	10%
Perennials	4727	85%	1722***	66%	336**	75%
*Total flora*	5574		2629		449	

*P<0.05; **P<0.01; ***P<0.001.

Percentages are relative to the total flora counts for each group. Native flora statistics do not include infraspecific taxa or hybrids. Bold counts are statistically significant outliers in Pearson chi-square analysis, comparing the alien flora counts with the native flora, and the invasive flora counts with the alien flora. Tests were performed separately for the three classifications indicated (specific growth forms, herbaceous vs. woody, and duration).

## Discussion

The typical alien vascular plant of the Eastern Deciduous Forest biome of the Eastern U.S. is a European forb, either from the Circumboreal northern and central regions of Europe or the southern Mediterranean region. The clear European bias in non-native plants has been documented in many global floras by plant biogeographers [6], [7], [29] and ecological historians [30], [31] and is referred to as the Imperialist Dogma [30]. This model asserts that the spread of European cultures since the Age of Discovery, including crops, weeds, and commensals, explains both the greater historical transport of European species to global floras and the greater ability of co-evolved European weeds to persist in landscapes dominated by agricultural practices that originated in southern Europe and the eastern Mediterranean [29], [30], [32]. When applied to all alien species, the Imperialist Dogma is supported in the present study by the biased representation of European species (including Circumboreal, Mediterranean, and Macronesian regions) in the non-native EUS flora; furthermore, the vast majority of invasive species in frequently disturbed habitats (weeds) stem from these regions. A central contribution of the present study, however, is to suggest that the Imperialist Dogma cannot be a general framework for plant invasions, because 1) alien species from Europe are less likely to be invasive than those from East Asia; 2) European species only dominate anthropogenic habitats such as managed agricultural areas, disturbed fields, waste places, and roadsides (Fig. 2)—and nonetheless as forests have greatly expanded in EUS over the past 150 years, plant invasions have increased; and 3) although alien species are typically European, the invasive flora is better described as Eurasian and is nearly as likely to come from central and east Asia as Europe (Table 2). Taken together, these observations suggest that the prevailing view of Europe as the ancestral cradle of plant invasions is only useful in so far as it describes the recent co-evolution of ‘weedy’ plants in historically novel human-dominated ecosystems [32]—a restricted set of conditions when viewed in the full context of plant invasions in a variety of disturbed and natural ecosystems worldwide.

In contrast to the total alien flora, EUS alien invaders are commonly woody species from East Asia, perhaps better reflecting EUS landscapes as dominated by closed secondary forests. Indeed, if the composition of the alien flora is used as a null model for invader composition, taxa from some regions are significantly more likely to invade (Table 2). It should come as no surprise that invasive taxa are most likely to come from areas with climates that resemble those of EUS (Fig. 1)—all floristic regions of greater-than-expected invader representation (>17%) are those of extra-tropical distribution. However, climate similarity is not sufficient to predict the bias in invader distribution among floristic regions. Part of this variance is attributable to native range size, in that species with native ranges that span continents are represented in many historically isolated floras, and native range size is well correlated with invasive potential [11], [33] (Fig. 3). This is particularly true of the high invader contribution of more arid temperate regions (including the Saharo-Arabian, Irano-Turanian, and Mediterranean)—regions with almost no EUS invaders endemic to them. Of particular interest is that, although overall those alien taxa endemic to particular floristic regions are very unlikely to be invaders (13%), those endemic to East Asia are nearly as invasive as the entire invader pool from East Asia (25% compared to 29%). Of those other regions with at least 20 EUS alien taxa that are natively endemic, only two—the Circumboreal and Chile-Patagonian regions—have at least 1 in 10 of those as invasive (11% and 10%, respectively), despite similar climates to the EUS existing on all continents [34].

Why is the flora—and in particular the woody forest flora—of East Asia so unusually invasive in the Eastern U.S.? From a broad historical perspective, colonization of EUS mesic forest habitat by East Asian plants is hardly novel. The late Pleistocene origin of the Eastern Deciduous Forest is thought largely to stem from the southern Appalachians and adjacent Cumberland Plateau [26], [35], and floristic similarities between this region and the forests of Japan and central China have been of great interest to botanists for centuries [36], [37]. These regions were connected via Beringia for much of the Tertiary, and taxonomic disjunctions, largely at the genus level, have resulted from periods of isolation following continental drift, increasing aridity in the Western U.S., and cool and dry conditions associated with major glaciation events in EUS [38], [39]. Interestingly, White [38] found these disjunct genera to be overrepresented by woody understory taxa, similar to the qualities of overrepresented invasive taxa reported here. Furthermore, most of the major woody forest EUS invaders endemic to East Asia have congeners in the EUS native flora, including *Berberis thunbergii* (native is *B. canadensis*), *Celastrus orbiculatus* (near-endemic to East Asia, native is *C. scandens*), *Elaeagnus umbellata* (*E. commutata*), *Euonymus alatus* (several native bush *Euonymus*), *Lonicera morrowii* (*L. canadensis*), *Rosa multiflora* (several natives), *Viburnum dilatatum* (several natives), and *Wisteria sinensis* (*W. futescens*), among others. It is therefore tempting to suggest that the modern invasion of EUS forests is only the latest chapter in a long history of highly (pre)adapted East Asian lineages colonizing mesic temperate forests worldwide. Consistent with this view, few if any woody understory species from EUS (or Europe) made a list of 126 non-native plant species in China [40]. If true, it suggests that forest invasion mechanisms can be deconstructed by comparative ecophysiological studies of East Asian-EUS sister taxa. It also qualitatively supports patterns of biotic interchanges throughout geologic history, in that modern invasions are similarly characterized by certain regions donating more invaders to particular habitats [2].

An important component of invasive species management is the prevention or early detection of species that exhibit strong invasive tendencies [41], and the association of invaders from certain regions with particular habitats (Fig. 2) suggests several guidelines for natural area management in the EUS. First, although the European bias in alien species persists for those invaders of open and managed habitats, European species are significantly less likely to pose significant management concern in forested natural areas of the Eastern U.S. Instead, managers should be particularly concerned about current and future introductions of woody plants from East Asia that already account for the majority of woody species that dominate forest understories. Second, native endemism can be an important tool for screening plant invasive potential. It is already well appreciated that species of larger native ranges are more likely to become pests in their introduced range [11], [42]; the present study confirms this and adds greater detail by classifying endemism according to specific regions. For example, although a significant number of EUS invaders are sub-Saharan African in origin (particularly warm-season grasses), there is not a single EUS invader endemic to an African floristic region (Table 2). On the other hand, there are only four EUS alien taxa endemic to the Indo-Chinese region of southeast Asia, and yet two of these are invasive, again attesting to the strong invasive potential of Asian taxa in the Eastern U.S. Finally, the strong bias toward woody plants in the invasive pool (39%) compared to the alien (23%) or native floras (24%), despite many invasive lists being derived from agricultural activities where woody species are less common, suggests conservationists and natural resource managers in the EUS focus energies on preventing the introduction and local establishment of non-native woody species [17], [43]. It is also important to consider that many alien species only become invasive after significant time lags [44], suggesting that Asian woody taxa considered non-invasive in the present study nonetheless be treated carefully in horticultural practice.

The clear floristic distinction between alien and invasive plant taxa in the EUS, and the strong biases in habitat representation between invaders of different origin and life history attributes, is further rationale for more careful delimitation of the focal species pool in invasion studies [23]. In particular, the failure of plant ecologists to identify easily screened attributes of ‘invaders’ should be expected if analyses include all alien (non-native, exotic) taxa [45]. Species that successfully naturalize do share attributes relating to long-distance dispersal ability and reproductive potential [7], [10], [11], but the present study suggests critical attributes of those that become invasive are specific to particular environmental circumstances rather than universal across habitat types. If other global regions show floristic biases in the invasive species pools of particular habitats that resemble those described in the present study, there should be renewed motivation for comparative studies of the biology of plants from different floras. As modern invasions increasingly appear to qualitatively resemble past biotic interchanges [3], [4], [5], such comparative studies may also help paleobiologists better understand the historical development of modern plant assemblages.

## Materials and Methods

I constructed a database (Supplemental Dataset S1, with associated metadata in Supplemental Text S1) of all alien vascular plant taxa present in the Eastern Deciduous Forest of the Eastern U.S. (state occurrences from MN to LA, east to the coasts of ME to GA, excluding presences unique to FL) using the USDA PLANTS database [46]. I defined as alien those taxa listed as “Introduced” by USDA PLANTS residing in the above states. Taxa such as *Phragmites australis* with native and exotic populations listed as “Native and Introduced” were not included. I included unambiguous non-native infraspecific taxa (e.g., *Taraxacum officinalis* ssp. *officinalis*, *Ranunculus acris* var. *acris*, *Viburnum opulus* var. *opulus*) that are tracked by PLANTS. Alien plant species in the U.S. are only tracked by PLANTS if their native range is wholly outside the contiguous U.S., preventing analysis of those alien taxa native to the Western U.S. Alien plants were categorized as “invasive” if they were represented on the USDA PLANTS “Weedy and Invasive Plants” lists for Eastern U.S. regions, including the Northeast [47], Kentucky [48], Tennessee/Southeast [49], and Wisconsin [50], plus any remaining alien taxa that were indicated as present in the selected states in the WeedsUS database maintained by the U.S. National Park Service [51]. The invasive plant definition used here is thus an alien in the Eastern U.S. of significant management concern.

All species were assigned growth form and duration attributes according to the USDA PLANTS database. Growth form attributes included the non-exclusive forms “tree”, “shrub” (including “subshrub”), “vine”, “graminoid”, and “forb/herb”; “herbaceous” and “woody” classes were derived from lumping “graminoid” and “forb/herb” forms (which includes all herbaceous vines) and “tree”, “shrub”, and “vine” forms (using only those vines which were not also listed as forbs). A small set of species are semi-woody and are included in herbaceous and woody categories. Duration attributes included annual, biennial, and perennial designations. The composition of the alien flora with respect to these attributes was compared to the EUS native flora using a species-level query of contiguous U.S.-native plants residing in the above selected states from PLANTS. The subset of alien species defined as invasive was further assigned habitat designations describing the environmental circumstances of their occurrences in EUS. Detailed habitat descriptions were first obtained from major EUS floras [52], [53], [54]; these idiosyncratic descriptions (e.g., “wet meadows”, “bottomland hardwood forests”) were then grouped into seven habitat classes meant to describe important environmental correlates (disturbance regime, light availability, soil moisture status). Table 1 summarizes this classification, as illustrated in Fig. 2. Non-invasive alien species are typically rare in their introduced ranges, preventing any reliable assessment of foreign habitat affinity for these taxa.

All alien taxa in the EUS flora were assigned membership to native source floras using the floristic region designations of Takhtajan [27] (Fig. 1). Takhtajan's system is based on geographic patterns of endemism, particularly at the species and genus levels, and is meant to represent patterns of historical isolation and evolutionary divergence in the global distribution of vascular plants [55]. Along with the antecedent work of Good [56], to which it closely coincides, it remains the only attempt to categorize the world's flora phylogenetically at the sub-continental scale [55]. For studies of plant species behavior based on aspects of their evolutionary history, Takhtajan's regions thus represent a clear advantage over native biogeographic units based on geopolitical boundaries. Each alien taxon was assigned to one or more Takhtajan regions according to documented native range descriptions from source floras. The majority of these assignments were accomplished with taxon queries in the online Germplasm Resources Information Network [57], a central location of floristic distribution information compiled from world floras. In some cases where GRIN records were unavailable, a number of other source floras were consulted. In general, the assignment of native ranges to floristic regions for those taxa distributed close to region boundaries was conservative. A list of native floristic regions for each taxon, along with additional bibliographic information, is available as Supplement Dataset S1 and Text S1. Due to small spatial resolution and sample sizes of alien taxa, three Takhtajan regions for the southern tip of Africa were combined into a single region, as were the three floristic regions of Australia (Table 2). Alien taxa were also essentially absent from small island or archipelago regions, and are ignored in the present analysis.

Contingency tables of floristic region vs. habitat and floristic region vs. growth form were analyzed for independence with Pearson chi-square tests in R [58]. Significant residuals were identified with the Freeman-Tukey deviate statistic [59], with a threshold of an expected count of at least 5 for significance [60].

## Supporting Information

Dataset S1 Native floristic zones of alien plant taxa of the Eastern U.S. database.
(0.92 MB XLS)

Text S1 Native floristic zones of alien plant taxa of the Eastern U.S. database: metadata.
(0.04 MB DOC)

## References

[1] Darwin C (1859). On the origin of species. Facsimile reproduction of the First Edition.

[2] Gentry AH (1982). Neotropical floristic diversity: phytogeographical connections between Central and South America, Pleistocene climatic fluctuations, or an accident of the Andean orogeny?. Ann Miss Bot Gar.

[3] Vermeij GJ (1996). An agenda for invasion biology.. Biological Conservation.

[4] Vermeij GJ, Sax DF, Gaines SD, Stachowicz JJ (2005). Invasion as expectation: a historical fact of life.. Species invasions: insights into ecology, evolution, and biogeography.

[5] Brown JH, Sax DF (2004). An essay on some topics concerning invasive species.. Aust Ecol.

[6] Groves RH, Groves RH, di Castri F (1991). The biogeography of Mediterranean plant invasions.. Biogeography of mediterranean invasions.

[7] Pyšek P (1998). Is there a taxonomic pattern to plant invasions?. Oikos.

[8] Perrings C, Dehnen-Schmutz K, Touza J, Williamson M (2005). How to manage biological invasions under globalization.. Trends Ecol Evol.

[9] Thompson K, Hodgson JG, Rich TCG (1995). Native and alien invasive plants: more of the same?. Ecography.

[10] Crawley MJ, Harvey PH, Purvis A (1996). Comparative ecology of the native and alien floras of the British Isles.. Phil Trans R Soc Lond B.

[11] Richardson DM, Pyšek P (2006). Plant invasions: merging the concepts of species invasiveness and community invasibility.. Progr Phys Geog.

[12] Crawley MJ, Gray AJ, Crawley MJ, Edwards PJ (1987). What makes a community invasible?. Colonization, succession and stability.

[13] Lonsdale WM (1999). Global patterns of plant invasions and the concept of invasibility.. Ecology.

[14] Davis MA, Grime JP, Thompson K (2000). Fluctuating resources in plant communities: a general theory of invasibility.. J Ecol.

[15] Fridley JD, Stachowicz JJ, Naeem S, Sax DF, Seabloom EW (2007). The invasion paradox: reconciling pattern and process in species invasions.. Ecology.

[16] Mack RN (2003b). Plant naturalizations and invasions in the Eastern United States: 1634–1860.. Ann Miss Bot Gar.

[17] Webster CR, Jenkins MA, Jose S (2006). Woody invaders and the challenges they pose to forest ecosystems in the Eastern United States.. J For.

[18] Williamson M, Fitter A (1996a). The varying success of invaders.. Ecology.

[19] Vilá M, Pujadas J (2001). Land-use and socio-economic correlates of plant invasions in European and North African countries.. Biol Cons.

[20] Peterson AT, Papes M, Kluza DA (2003). Predicting the potential invasive distributions of four alien plant species in North America.. Weed Sci.

[21] Mack RN (2003a). Phylogenetic constraint, absent life forms, and preadapted alien plants: a prescription for biological invasions.. Int'l J Plant Sci.

[22] Williamson M, Fitter A (1996b). The characters of successful invaders.. Biol Cons.

[23] Daehler CC (1998). The taxonomic distribution of invasive angiosperm plants: ecological insights and comparison to agricultural weeds.. Biol Cons.

[24] Hobbs RJ, Huenneke LF (1992). Disturbance, diversity, and invasion: implications for conservation.. Cons Biol.

[25] Grime JP (2001). Plant strategies, vegetation processes, and ecosystem properties. Second edition.

[26] Braun EL (1950). Deciduous forests of Eastern North America.

[27] Takhtajan A (1986). Floristic regions of the world.

[28] Cassey P, Blackburn TM, Duncan RP, Chown SL (2005). Concerning invasive species: reply to Brown and Sax.. Aust Ecol.

[29] Heywood VH, Drake JA (1989). Patterns, extents and modes of invasion by terrestrial plants.. Biology invasions: a global perspective.

[30] Crosby AW (1986). Ecological imperialism: the biological expansion of Europe, 900–1900.

[31] Diamond J (1999). Guns, germs, and steel: the fates of human societies.

[32] di Castri F, Drake JA (1989). History of biology invasions with special emphasis on the Old World.. Biology invasions: a global perspective.

[33] Goodwin BJ, McAllister AJ, Fahrig J (1999). Predicting invasiveness of plant species based on biological information.. Cons Biol.

[34] Kottek M, Grieser J, Beck C, Rudolf B, Rubel F (2006). World map of Köppen-Geiger climate classification updated.. Meteorologische Zeitschrift.

[35] Whittaker RH (1956). Vegetation of the Great Smoky Mountains.. Ecol Monogr.

[36] Li H-L (1952). Floristic relation between eastern Asia and eastern North America.. Trans Amer Philos Soc.

[37] Boufford DE, Spongberg SA (1983). Eastern Asian-eastern North American phytogeographical relationships: a history from the time of Linnaeus to the twentieth century.. Ann Miss Bot Gar.

[38] White PS (1983). Eastern Asian-Eastern North American floristic relations: the plant community level.. Ann Miss Bot Gar.

[39] Qian H (2002). Floristic relationships between Eastern Asia and North America: test of Gray's hypothesis.. Am Nat.

[40] Liu J, Dong M, Miao SL, Li ZY, Song MH (2006). Invasive alien plants in China: role of clonality and geographical origin.. Biol Inv.

[41] Ruesink JL, Parker IM, Groom MJ, Karieva PM (1995). Reducing the risks of nonindigenous species introductions.. Bioscience.

[42] Rejmánek M (1996). A theory of seed plant invasiveness: the first sketch.. Biol Cons.

[43] Binggeli P (1996). A taxonomic, biogeographical and ecological overview of invasive woody plants.. J Veg Sci.

[44] Salisbury E (1961). Weeds and aliens.

[45] Pyšek P, Richardson DM, Rejmánek M, Webster GL, Williamson M (2004). Alien plants in checklists and floras: towards better communication between taxonomists and ecologists.. Taxon.

[46] USDA, NRCS (2007). The PLANTS Database.. http://plants.usda.gov.

[47] Uva RH, Neal JC, DiTomaso JM (1997). Weeds of the Northeast.

[48] Haragan PD (1991). Weeds of Kentucky and adjacent states: a field guide.

[49] Southeast Exotic Pest Plant Council (1996). Invasive Exotic Pest Plants in Tennessee (19 October 1999).

[50] Hoffman R, Kearns K (1997). Wisconsin manual of control recommendations for ecologically invasive plants.

[51] Swearingen J (2008). WeedUS: Database of plants invading natural areas in the United States.. http://www.nps.gov/plants/alien/list/WeedUS.xls.

[52] Gleason HA, Cronquist A (1991). Manual of vascular plants of northeastern United States and adjacent Canada.

[53] Weakley AS (2008). Flora of the Carolinas, Virginia, and Georgia, and surrounding areas. April 2008 version.

[54] Flora of North America Editorial Committee, editors (1993+). Flora of North America North of Mexico. 12+ vols.

[55] Cox CB (2001). The biogeographic regions reconsidered.. J Biogeog.

[56] Good R (1947, 1953, 1964, 1974). The geography of flowering plants. Editions 1–4.

[57] USDA, ARS, National Genetic Resources Program (2007). Germplasm Resources Information Network - (GRIN).. http://www.ars-grin.gov/cgi-bin/npgs/html/paper.pl?language=en.

[58] R Development Core Team (2006). R: A language and environment for statistical computing.

[59] Sokal RR, Rohlf SJ (1995). Biometry.

[60] Legendre P, Legendre L (1998). Numerical ecology. Second Edition.

