# Supplementary material for: Of Asian Forests and European Fields: Eastern U.S. Plant Invasions in a Global Floristic Context
Source: PLoS One. 2008 Nov 3;3(11):e3630. doi: 10.1371/journal.pone.0003630 (PMC2572842; doi:10.1371/journal.pone.0003630)
Supplement: Text S1 — Native floristic zones of alien plant taxa of the Eastern U.S. database: metadata. (0.04 MB DOC) [file pone.0003630.s002.doc]

Supplement Text S1: Native floristic zones of alien plant taxa of the Eastern U.S. database: metadata.

**First nine columns** are from USDA PLANTS (2007), including USDA_code, Scientific Name, State Distribution, Family, Order, Class, Division, GrowthHabit, Duration, Nativity.

**Native floristic regions (Takhtajan)**: Occurrence of the native distribution of a taxon in one or more global floristic regions as defined by Takhtajan (1986). Numbers refer to one of Takhtajan's 35 regions; see Table 2.

**Floristic region source**: Source of native distributional information. Abbreviated sources and their citations include:

GRIN = USDA, ARS (2007)

FNA = FNA Editorial Committee (1993+)

Flora of China = Wu and Raven (1994+)

Flora of Japan = Ohwi et al. (1965)

Flora of Pakistan = Nasir and Ali (1971)

Flora Europea = Tutin et al. (1964-1980)

Flora USSR = Komarov (1934-64)

PLANTS = USDA, NRCS (2007)

Flora der Schweiz = Hess (1967)

TROPICOS = Missouri Botanical Garden (2007)

Australia Virtual Herbarium = Centre for Plant Biodiversity Research (2003)

**Floristic region comments**: notes on difficulties with distribution or taxon.

**Invasive in ENA**: source of 'invasive' designation, where applicable:

USWEEDS = Swearingen 2008

WI = Hoffman and Kearns 1997

SEEPPC = Southeast Exotic Pest Plant Council 1996

N'EAST = Uva et al. 1997

KY = Haragan 1991

**Habitat classes (invasive only)**: association of taxon with habitat classes as defined in Table 1.

**Detailed habitat description (invasive only)**: obtained from Eastern U.S. floras, including Gleason and Cronquist 1991, FNA Editorial Committee 1993+, Weakley 2008.

Supplement References

Centre for Plant Biodiversity Research (2007) Australia's Virtual Herbarium [map output]. Council of Heads of Australian Herbaria. Available at http://www.cpbr.gov.au/cgi-bin/avh.cgi. Accessed 2007.

Flora of North America Editorial Committee, editors (1993+) Flora of North America North of Mexico. 12+ vols. New York and Oxford.

Gleason HA, Cronquist A (1991) Manual of vascular plants of northeastern United States and adjacent Canada. Bronx: New York Botanical Garden.

Haragan PD (1991) Weeds of Kentucky and adjacent states: a field guide*.* Lexington: University Press of Kentucky.

Hess HE (1967) Flora der Schweiz und angrenzender Gebiete. Basel: Birkhaüser.

Hoffman R, Kearns K, editors (1997) Wisconsin manual of control recommendations for ecologically invasive plants. Madison: Wisconsin Dept. Natural Resources.

Komarov VL, editor (1934–1964) Flora of the USSR. Translated from the Russian by the Israel Program for Scientific Translations for the Smithsonian Institute.

Missouri Botanical Garden (2007) TROPICOS: online database. Available at http://www.tropicos.org/. Accessed 2007.

Nasir E, Ali SJ (1971) Flora of Pakistan. St. Louis: University of Karachi & Missouri Botanical Garden. Accessed online via eFloras: http://www.efloras.org/flora_page.aspx?flora_id=2. Accessed 2007.

Ohwi J, Meyer FG, Walker EH (1965) Flora of Japan. Washington: Smithsonian Institution.

Southeast Exotic Pest Plant Council (1996) Invasive Exotic Pest Plants in Tennessee (19 October 1999). Tennessee: Research Committee of the Tennessee Exotic Pest Plant Council.

Swearingen J (2008) WeedUS: Database of plants invading natural areas in the United States. Available: http://www.nps.gov/plants/alien/list/WeedUS.xls. Accessed May 3, 2007.

Takhtajan A (1986) Floristic regions of the world. Berkeley: University of California Press.

Tutin TG et al., editors (1964–1980) Flora Europaea. Available at http://rbg-web2.rbge.org.uk/FE/fe.html (Royal Botanic Garden Edinburgh). Accessed 2007.

USDA, ARS, National Genetic Resources Program (2007) Germplasm Resources Information Network - (GRIN). National Germplasm Resources Laboratory, Beltsville, Maryland. Available at http://www.ars-grin.gov/cgi-bin/npgs/html/paper.pl?language=en. Accessed 2007.

USDA, NRCS (2007) The PLANTS Database. National Plant Data Center, Baton Rouge, LA. Available at http://plants.usda.gov. Accessed 2007.

Uva RH, Neal JC, DiTomaso JM (1997) Weeds of the Northeast*.* Ithaca: Cornell University Press.

Weakley AS (2008) Flora of the Carolinas, Virginia, and Georgia, and surrounding areas. April 2008 version. Chapel Hill: UNC Herbarium, North Carolina Botanical Garden, University of North Carolina at Chapel Hill.

Wu Z, Raven PH, editors (1994+) Flora of China. St. Louis: Science Press, Missouri Botanical Garden. Available via eFloras: http://www.efloras.org/flora_page.aspx?flora_id=2. Accessed 2007.
